# Seroprevalence of IgG antibodies against diphtheria antitoxin among migrant workers in Singapore, 2016–2019

**DOI:** 10.1186/s12889-022-12528-y

**Published:** 2022-01-16

**Authors:** Li Wei Ang, Qi Gao, Lin Cui, Aysha Farwin, Matthias Paul Han Sim Toh, Irving Charles Boudville, Mark I-Cheng Chen, Angela Chow, Raymond Tzer-Pin Lin, Vernon Jian Ming Lee, Yee Sin Leo

**Affiliations:** 1 National Public Health and Epidemiology Unit, National Centre for Infectious Diseases, 16 Jalan Tan Tock Seng, Singapore, 308442 Singapore; 2 Public Health Group, Ministry of Health, Singapore, Singapore; 3 National Public Health Laboratory, National Centre for Infectious Diseases, Singapore, Singapore; 4 Saw Swee Hock School of Public Health, National University of Singapore, Singapore, Singapore; 5 Lee Kong Chian School of Medicine, Nanyang Technological University, Singapore, Singapore; 6 Department of Clinical Epidemiology, Office of Clinical Epidemiology, Analytics, and Knowledge (OCEAN), Tan Tock Seng Hospital, Singapore, Singapore; 7 Yong Loo Lin School of Medicine, National University of Singapore, Singapore, Singapore; 8 Department of Laboratory Medicine, National University Hospital, National University Health System, Singapore, Singapore; 9 National Centre for Infectious Diseases, Singapore, Singapore; 10 Department of Infectious Diseases, Tan Tock Seng Hospital, Singapore, Singapore

**Keywords:** Diphtheria, Immunity, Seroprevalence, Vaccination coverage, Basic protection, Migrant workers

## Abstract

**Background:**

Since the last local case of diphtheria in 1992, there had not been any case in Singapore until an autochthonous case was reported in 2017. This fatal diphtheria case of a migrant worker raised concerns about the potential re-emergence of locally transmitted toxigenic diphtheria in Singapore. We conducted a seroprevalence study to assess the immunity levels to diphtheria among migrant workers in Singapore.

**Methods:**

Residual sera from migrant workers who hailed from Bangladesh, China, India, Indonesia, Malaysia, Myanmar and the Philippines were tested for anti-diphtheria toxoid immunoglobulin G (IgG) antibodies. These migrant workers previously participated in a survey between 2016 and 2019 and had provided blood samples as part of the survey procedure.

**Results:**

A total of 2176 migrant workers were included in the study. Their overall mean age was 27.1 years (standard deviation 5.0), range was 20–43 years. The proportion having at least basic protection against diphtheria (antitoxin titres ≥ 0.01 IU/ml) ranged from 77.9% (95% confidence interval [CI] 72.8 – 82.3%) among migrant workers from Bangladesh to 96.7% (95% CI 92.5 – 98.6%) in those hailing from Malaysia. The proportion showing full protection (antitoxin titres ≥ 0.10 IU/ml) ranged from 10.1% (95% CI 6.5 – 15.4%) in Chinese workers to 23.0% (95% CI 17.1 – 30.3%) in Malaysian workers. There were no significant differences in the proportion with at least basic protection across birth cohorts, except for those from Bangladesh where the seroprevalence was significantly lower in younger migrant workers born after 1989.

**Conclusions:**

The proportions having at least basic protection against diphtheria in migrant workers from five out of seven Asian countries (India, Indonesia, Malaysia, Myanmar and the Philippines) were higher than 85%, the threshold for diphtheria herd immunity. Seroprevalence surveys should be conducted periodically to assess the level of immunity against diphtheria and other vaccine preventable diseases in migrant worker population, so that appropriate interventions such as booster vaccination can be implemented proactively to prevent sporadic outbreaks.

**Supplementary Information:**

The online version contains supplementary material available at 10.1186/s12889-022-12528-y.

## Introduction

Diphtheria is a severe bacterial infection caused by toxin-producing strains of *Corynebacterium diphtheriae*. Complications of respiratory diphtheria require early detection, prompt treatment with diphtheria antitoxin and antibiotics, and intensive care interventions in severe cases. The overall case-fatality ratio (CFR) for diphtheria ranges from 5 to 10%, with higher CFR in children aged < 5 years and adults > 40 years [1].

Although there has been a drastic reduction in morbidity and mortality after the introduction of diphtheria toxoid-containing vaccine, diphtheria remains a public health issue particularly in areas with low vaccination coverage [2]. In order to confer diphtheria herd immunity, at least 85% of each birth cohort needs to be vaccinated [3]. According to a World Health Organization (WHO) manual for management and control of diphtheria, a minimum immunity rate of 90% in children and 75% in adults is required for diphtheria elimination [4]. However, knowledge gaps about the epidemiology, transmission and control of diphtheria exist due to the lack of attention to this disease in the last century [5]. Inadequate homogeneous coverage with three doses of diphtheria toxoid-containing vaccine across countries and populations, and waning vaccine immunity in adults have resulted in recent resurgences of diphtheria [2]. Respiratory diphtheria outbreaks occurred in Indonesia, Bangladesh, Myanmar, Vietnam, Venezuela, Haiti, South Africa and Yemen in 2016–2019 [6], highlighting the need to identify susceptible population subgroups originating from these endemic or epidemic regions.

In Singapore, vaccination against diphtheria has been made compulsory by law for children under the National Childhood Immunisation Programme since 1962, with coverage for the primary course of diphtheria, tetanus, and acellular pertussis vaccine hovering between 95 and 97% in Singaporean children at 2 years of age since 2003 [7, 8]. A serological survey found that 92.0% of Singapore residents aged 18–79 years had at least basic protection against diphtheria (antibody levels ≥0.01 IU/ml) in 2010 [9]. However, seroprevalence studies assessing the diphtheria toxin antibody levels among subgroups of foreigners who may require additional vaccination efforts are lacking in Singapore. The foreign workforce comprised about a quarter of Singapore’s population of 5.69 million people as of December 2020, including 0.85 million work permit holders who mainly engage in semi-skilled work [10]. These latter group of migrant workers largely hail from China, Bangladesh, India, Malaysia, the Philippines, Myanmar and Thailand [11].

Since the last local case of diphtheria in 1992 [12], there had not been any local cases until an autochthonous case who died of respiratory obstruction was reported in 2017 [13]. The fatal diphtheria case was a 21-year-old male Bangladeshi construction worker who had been working in Singapore for the past 10 months [14]. This raised concerns about the potential re-emergence of locally transmitted toxigenic diphtheria in Singapore, particularly among migrant workers residing in dormitories who originate from countries with previously low childhood vaccination coverage. As an added precautionary measure, all contacts of the fatal diphtheria case with unknown vaccination history or who had not received vaccination in the previous five years were given a diphtheria toxoid booster vaccination [13].

The potential risk of diphtheria outbreaks provides the impetus for determining the immune status in at-risk populations for planning of outbreak prevention and control programs. To this end, we undertook a seroprevalence study to estimate the immunity levels to diphtheria among migrant workers in Singapore.

## Methods

Residual sera collected between 2016 and 2019 from a survey on latent tuberculosis among migrant workers in Singapore were used for our seroprevalence study in accordance with the Infectious Diseases Act [15], which provides for the use of residual blood samples for the purpose of public health surveillance.

The survey on latent tuberculosis involved migrant workers from eight Asian countries that had large number of workers in Singapore (Bangladesh, China, India, Indonesia, Malaysia, Myanmar, the Philippines, and Vietnam) [16]. They were recruited from 27 locations around Singapore, including clinics providing health screening services for migrant workers (67%), worker dormitories (30%) and recreation centers catering to migrant workers (3%). These migrant workers were between the ages of 20 and 50 years, had not previously worked in Singapore and had stayed here for less than a year. A total of 3584 migrant workers were included in the analysis for the survey on latent tuberculosis.

For this seroprevalence study, residual sera were available from 2191 migrant workers who participated in the survey on latent tuberculosis and provided full consent and partial consent for their data and residual sera to be used for future research. There was published data on seroprevalence of IgG antibodies against diphtheria antitoxin in Bangladesh [17], China [18], Indonesia [19] and Malaysia [20] (Table S1). On the premise of an anticipated prevalence of at least 50% having at least basic protection against diphtheria with a confidence level of 95% and an absolute precision of 8%, the minimum sample size required for migrant workers from each of the seven countries was 150. Only 15 migrant workers hailing from Vietnam had residual sera leftover for testing, hence they were excluded from the analysis.

Diphtheria toxoid IgG-specific antibody levels were measured using a commercial Anti-Diphtheria Toxoid Enzyme Immunoassay (EIA) (IgG) (Euroimmun, Germany). We followed guidelines from WHO [21] for this study and defined three levels of diphtheria antitoxin titres: < 0.01 IU/ml denotes susceptibility, 0.01–0.099 IU/ml denotes basic protection (i.e. giving basic immunity) and ≥ 0.10 IU/ml denotes full protection.

We used the Wilson method [22] to compute the 95% confidence interval (CI) for binomial proportions. The Mantel-Haenszel chi-square test for trend was used to evaluate the difference in seroprevalence across birth cohorts. Fisher’s exact test was used to test for differences in seroprevalence by gender. The geometric mean titre (GMT) of positive sera (antitoxin titres ≥ 0.01 IU/ml) and corresponding 95% CI were computed by first taking the logarithmic transformation of the titre readings, followed by antilog transformation of the mean and its 95% CI. We compared GMT by age group by computing the mean and 95% CI of the difference in logarithm-transformed antibody titres, followed by checking whether the ratio of 1 was within the confidence limits which had been antilog-transformed [23]. All analyses were performed using SPSS version 24 (IBM, USA). All *p* values reported were 2-sided and statistical significance was taken as *p* < 0.05.

## Results

A total of 2176 residual sera of migrant workers from seven Asian countries were included in the study; 672 (30.8%) from India, 434 (20.0%) from Indonesia, 289 (13.3%) from Bangladesh, 284 (13.0%) from Myanmar, 178 (8.2%) from China, 167 (7.7%) from the Philippines and 152 (7.0%) from Malaysia (Table 1). Men comprised the vast majority of migrant workers from Bangladesh (100%), China (93.9%), India (98.4%) and Malaysia (94.1%) (Table 2). More than 80% of migrant workers who hailed from Indonesia (99.8%), the Philippines (94.0%) and Myanmar (83.5%) were women. The overall mean age of migrant workers from the seven countries was 27.1 years (standard deviation 5.0), range was 20–43 years.

**Table 1:** Number and proportion of migrant workers in Singapore with at least basic protection against diphtheria (antitoxin titres ≥ 0.01 IU/ml) by country of origin and birth cohort

Country of origin [Total no.]	Birth cohort	No. of migrant workers	No. (%) with at least basic protection	*p*-value^╪^
Bangladesh [289]	1975–1984	31	29 (93.5)	0.001
	1985–1989	63	57 (90.5)	
	1990–1994	136	96 (70.6)	
	1995–1999	59	43 (72.9)	
China [178]	1975–1984	61	46 (75.4)	0.254
	1985–1989	65	55 (84.6)	
	1990–1994	41	35 (85.4)	
	1995–1999	11	9 (81.8)	
India [672]	1975–1984	98	93 (94.9)	0.514
	1985–1989	170	165 (97.1)	
	1990–1994	205	189 (92.2)	
	1995–1999	199	189 (95.0)	
Indonesia [434]	1975–1984	129	118 (91.5)	0.406
	1985–1989	101	95 (94.1)	
	1990–1994	204	182 (89.2)	
Malaysia [152]	1975–1984	18	17 (94.4)	0.468
	1985–1989	24	23 (95.8)	
	1990–1994	64	62 (96.9)	
	1995–1999	46	45 (97.8)	
Myanmar [284]	1975–1984	24	24 (100.0)	0.050
	1985–1989	48	45 (93.8)	
	1990–1994	207	180 (87.0)	
	1995–1999	5	5 (100.0)	
Philippines [167]	1975–1984	51	49 (96.1)	0.144
	1985–1989	61	58 (95.1)	
	1990–1994	55	49 (89.1)	

^╪^Chi-square test for trend across birth cohorts comparing the distribution for those with and without basic protection against diphtheria

**Table 2:** Number and proportion of migrant workers in Singapore with at least basic protection against diphtheria (antitoxin titres ≥ 0.01 IU/ml) by country of origin and gender

Country of origin [total no.]	Gender	No. of migrant workers	No. (%) with at least basic protection against diphtheria	*p*-value^╪^
Bangladesh [289]	Male	289	225 (77.9)	–
	Female	0	–	
China [178]	Male	167	134 (80.2)	0.221
	Female	11	11 (100.0)	
India [672]	Male	661	626 (94.7)	0.457
	Female	11	10 (90.9)	
Indonesia [434]	Male	1	0 (0.0)	–
	Female	433	395 (91.2)	
Malaysia [152]	Male	143	140 (97.9)	0.029
	Female	9	7 (77.8)	
Myanmar [284]	Male	47	42 (89.4)	1.000
	Female	237	212 (89.5)	
Philippines [167]	Male	10	10 (100.0)	1.000
	Female	157	146 (93.0)	

^╪^Fisher’s exact test for comparing the distribution by gender for those with and without basic protection against diphtheria

**Fig. 1:**
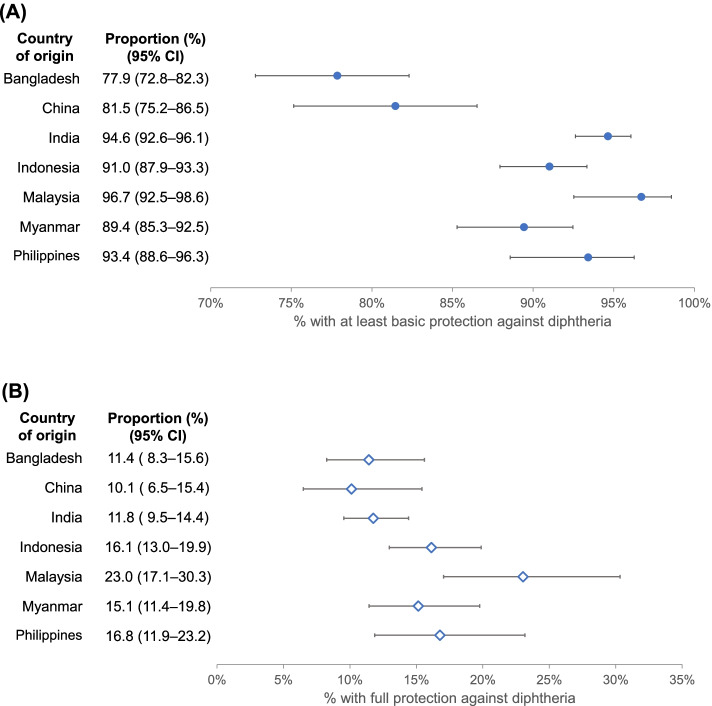
Overall percentage of migrant workers with (**A**) at least basic protection against diphtheria (antitoxin titres ≥ 0.01 IU/ml) (**B**) full protection against diphtheria (antitoxin titres ≥ 0.10 IU/ml), by country of origin. The error bars indicate 95% confidence interval (CI)

The country-specific proportion with at least basic protection against diphtheria (antitoxin titres ≥ 0.01 IU/ml) ranged from 77.9% (95% CI 72.8 – 82.3%) among migrant workers from Bangladesh to 96.7% (95% CI 92.5 – 98.6%) in those hailing from Malaysia (Fig. 1A). The proportion showing full protection (antitoxin titres ≥ 0.10 IU/ml) ranged from 10.1% (95% CI 6.5 – 15.4%) in Chinese workers to 23.0% (95% CI 17.1 – 30.3%) in Malaysian workers (Fig. 1B).

There were no significant differences in seroprevalence across birth cohorts among migrant workers from each Asian country, except for those from Bangladesh where the proportion with at least basic protection against diphtheria was significantly lower among younger migrant workers born after 1989 (Table 1). The seroprevalence of diphtheria in each birth cohort was above 85%, except for Bangladeshi migrant workers born in 1990–1999, and Chinese migrant workers born in 1975–1989 and 1995–1999.

The proportion having at least basic protection against diphtheria in male migrant workers from Malaysia was significantly higher than that of female counterparts (97.9% vs. 77.8%, *p* = 0.029), whereas no differences in gender-specific seroprevalence were observed in migrant workers from the other countries (Table 2).

**Fig. 2:**
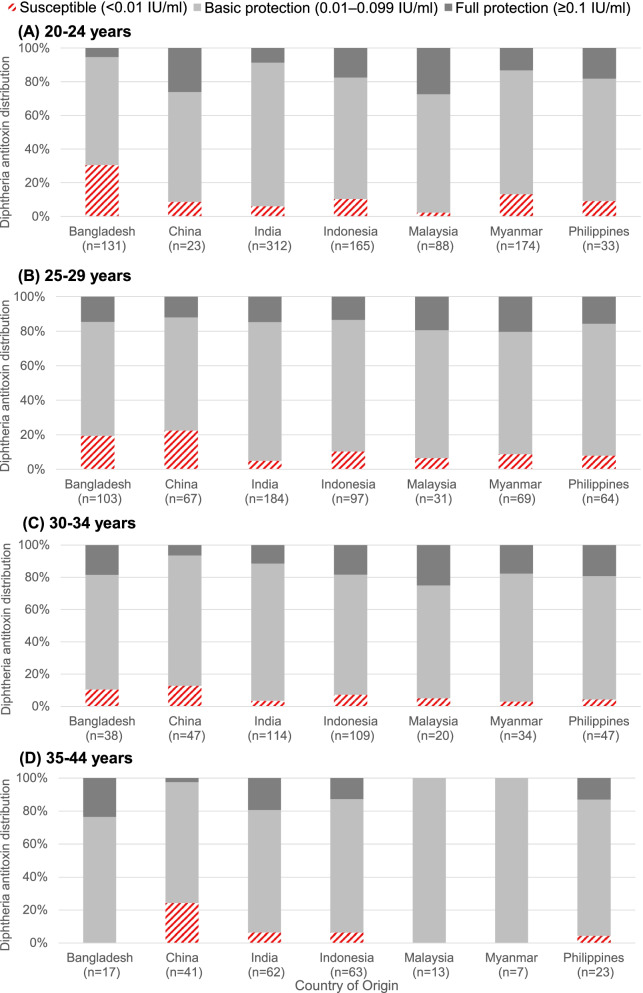
Distribution of diphtheria antitoxin titres (%) by country of origin and age group

The highest proportion susceptible to diphtheria (< 0.01 IU/ml) was observed in Bangladeshi migrant workers aged 20–24 years (30.5%), followed by Chinese workers aged 35–44 years (24.4%) and 25–29 years (22.4%), and Bangladeshi workers aged 25–29 years (19.4%) (Fig. 2). While 13.2 to 27.3% of migrant workers from Malaysia and Myanmar in the younger age groups (< 35 years) had full protection against diphtheria (≥0.10 IU/ml), there was none with full protection among those aged 35–44 years from these two countries. The proportion having full protection was lowest in the age group of 35–44 years for migrant workers from China, Indonesia and the Philippines. Conversely, 23.5% of migrant workers from Bangladesh and 19.4% from India in the 35–44 years age group had full protection and these were the highest proportion for the two countries.

**Fig. 3:**
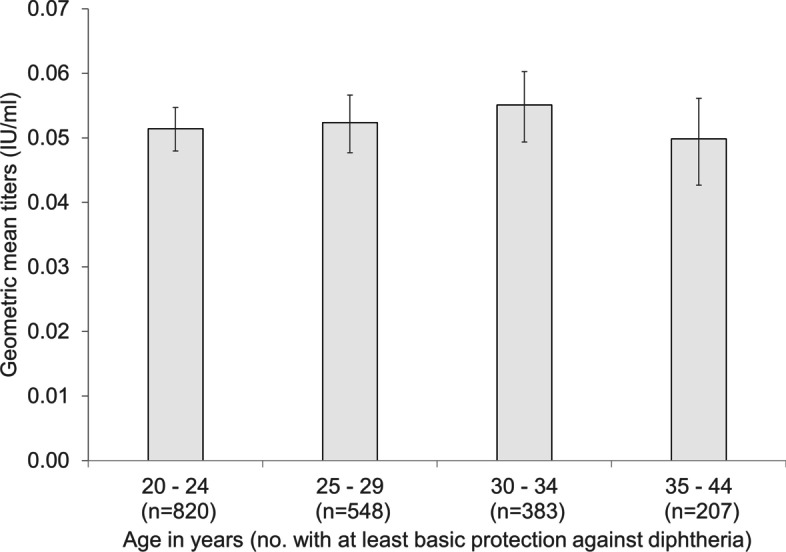
Geometric mean titres of diphtheria toxoid IgG antibodies in seropositive migrant workers with at least basic protection against diphtheria (≥ 0.01 IU/ml) by age group

Among seropositive migrant workers with at least basic protection (≥ 0.01 IU/ml), those aged 35–44 years had the lowest GMT of diphtheria toxoid IgG antibodies of 0.050 IU/ml (95% CI 0.044–0.057) (Fig. 3), however differences between age groups were not statistically significant.

## Discussion

The proportion having at least basic protection against diphtheria (≥ 0.01 IU/ml) was higher than the threshold of 85% for diphtheria herd immunity [24, 25] in migrant workers from India, Indonesia, Malaysia, Myanmar and the Philippines. Although the point estimates of the seroprevalence of diphtheria toxoid IgG antibodies were lower among migrant workers from Bangladesh and China, both exceeded 75%, the threshold indicated by Dadswell as sufficient to prevent an outbreak of diphtheria [26].

Diphtheria is endemic in all the seven Asian countries where the migrant workers hailed from [27]. Migrant workers originating from diphtheria endemic or epidemic regions pose a risk of introducing this disease upon their entry into Singapore. The humoral immunity against diphtheria seen in migrant workers from each of these countries may be possibly due to natural infection rather than vaccination for those in the older age groups. In 1991, the diphtheria tetanus toxoid and pertussis (DTP3) vaccination coverage among 1-year-olds ranged from 57% in India to 94% in China (Table S2). In 2019, the DTP3 vaccination coverage had all shown an increase in these Asian countries except in the Philippines.

Most of the diphtheria cases reported globally since 2000 have come from the WHO South-East Asia region [28]. Over a 10-year period from 2007 to 2016, India reported the highest incidence rate of diphtheria cases among the seven Asian countries where the migrant workers came from (Table S2). A global review of recent epidemiologic trends found that a higher proportion of diphtheria cases were below 15 years of age and unvaccinated in countries with high number of cases, whereas there was a shift to older age in countries with sporadic cases indicating waning vaccine immunity [28]. The lower proportion of full protection against diphtheria in migrant workers aged 35–44 years compared with their younger counterparts from five out of seven countries in our study was consistent with the decline in antitoxin levels over time. Although some may still be protected in adulthood, the majority maintain only minimal protective antitoxin levels 10 years after the last dose due to waning antitoxin titres [29]. Due to a previous lack of global guidance on diphtheria-containing booster doses after the 3-dose primary course, vaccination schedules vary widely in different countries [28]. In 2017, WHO modified their position on adult vaccinations and recommended vaccinating adults against tetanus and diphtheria only if they did not complete their childhood vaccination series or did not know whether they did [30].

This study provides complementary surveillance information on immunity against diphtheria among migrant workers in Singapore, and sheds light on the effectiveness of vaccination programs in the countries where they originated from. Data on the distribution of migrant workers by country of origin is unavailable, hence we are unable to assess whether our study sample was representative of the migrant worker population in Singapore. Furthermore, this study was based on residual sera from a previous survey. Another caveat is the small number of migrant workers in some age groups, hence the breakdown of results by country of origin and age group warrants careful interpretation. Nevertheless, this study sheds light on the susceptibility of migrant workers to diphtheria in Singapore.

In view of the varying proportion of migrant workers with basic protection against diphtheria coupled with the expectation of waning immunity with age, it is crucial to maintain a high degree of vigilance. Periodic seroprevalence surveys are needed to assess the level of immunity against diphtheria and other vaccine preventable diseases in the migrant worker populations, so that appropriate interventions such as booster vaccination can be implemented proactively to prevent sporadic outbreaks.

## Supplementary Information

**Additional file 1: Supplementary Table 1.** Seroprevalence of IgG antibodies against diphtheria antitoxin in Bangladesh, China, Indonesia and Malaysia. **Supplementary Table 2.** Diphtheria tetanus toxoid and pertussis (DTP3) vaccination coverage (%) among 1-year-olds and incidence rate per million population of diphtheria cases in Bangladesh, China, India, Indonesia, Malaysia, Myanmar and the Philippines.

## Data Availability

The data that support the findings of this study are available from Qi Gao, National Public Health and Epidemiology Unit, but restrictions apply to the availability of the data, which was used under license for the current study, and so are not publicly available. Data are however available from the authors upon reasonable request.

## Abbreviations

CFR: Case-fatality ratio
CI: Confidence interval
EIA: Enzyme Immunoassay
GMT: Geometric mean titre
IgG: Immunoglobulin G
WHO: World Health Organization
