# Supplementary material for: Seroprevalence of IgG antibodies against diphtheria antitoxin among migrant workers in Singapore, 2016–2019
Source: BMC Public Health. 2022 Jan 16;22:111. doi: 10.1186/s12889-022-12528-y (PMC8761332; doi:10.1186/s12889-022-12528-y)
Supplement: Supplementary file 1 — Additional file 1: Supplementary Table 1. Seroprevalence of IgG antibodies against diphtheria antitoxin in Bangladesh, China, Indonesia and Malaysia. Supplementary Table 2. Diphtheria tetanus toxoid and pertussis (DTP3) vaccination coverage (%) among 1-year-olds and incidence rate per million population of diphtheria cases in Bangladesh, China, India, Indonesia, Malaysia, Myanmar and the Philippines. [file 12889_2022_12528_MOESM1_ESM.pdf]

Seroprevalence of IgG antibodies against diphtheria antitoxin among migrant workers in Singapore, 2016–2019

Li Wei Ang, Qi Gao, Lin Cui, Aysha Farwin, Matthias Paul Han Sim Toh, Irving Charles Boudville, Mark I-Cheng Chen, Angela Chow, Raymond Tzer-Pin Lin, Vernon Jian Ming Lee, Yee Sin Leo

Supplementary Table 1. Seroprevalence of IgG antibodies against diphtheria antitoxin in Bangladesh, China, Indonesia and Malaysia

Country	Period of survey	Study group	Diphtheria seroprevalence	
			Antitoxin titres ≥0.01 IU/ml	Antitoxin titres ≥0.1 IU/ml
Bangladesh [1]	2018	930 children aged 6 months – 14 years in Nayapara and makeshift settlements (MSs)	88% in 1–6 year-olds and 93% in 7–14 year-olds in MSs 99% in 1–6 year-olds in Nayapara	63% in 1–6 year-olds and 77% in 7–14 year-olds in MSs, 91% in 1–6 year-olds in Nayapara
China [2]	2012	2147 residents aged ≤74 years in Beijing	-	52.5% in 25–29 year-olds, 43.7% in 30–34 year-olds, 37.4% in 35–39 year-olds
Indonesia [3]	2012	290 children aged 1–15 years in East Java; 147 in low incidence district of Kediri on the mainland of East Java, 143 in high incidence district of Bangkalan on the island of Madura	93% in Kediri, 90% in Bangkalan	71% in Kediri, 83% in Bangkalan
Malaysia [4]	January to July 2008	152 medical students and staff in Universiti Putra Malaysia	100%	77.6%

References

[1] Feldstein LR, Bennett SD, Estivariz CF, Cooley GM, Weil L, Billah MM, Uzzaman MS, Bohara R, Vandenent M, Adhikari JM, Leidman E, Hasan M, Akhtar S, Hasman A, Conklin L, Ehlman D, Alamgir A, Flora MS. Vaccination coverage survey and seroprevalence among forcibly displaced Rohingya children, Cox's Bazar, Bangladesh, 2018: A cross-sectional study. PLos Med. 2020;17(3): e1003071. doi: 10.1371/journal.pmed.1003071.

[2] Li X, Chen M, Zhang T, Li J, Zeng Y, Lu L. Seroepidemiology of diphtheria and pertussis in Beijing, China: A cross-sectional study. Hum Vaccin Immunother. 2015;11(10):2434-9. doi: 10.1080/21645515.2015.1062954.

[3] Hughes G, Mikhail AFW, Husada D, Irawan E, Kafatos G, Bracebridge S, Pebody R, Efstratiou A. Seroprevalence and determinants of immunity to diphtheria for children living in two districts of contrasting incidence during an outbreak in East Java, Indonesia. Pediatr Infect Dis J. 2015;34(11): 1152-1156. doi: 10.1097/INF.0000000000000846.

[4] Hamat RA, Malina O, Chua YJ, Seng KL, Zubaidah M, Norhanim K, Chong SS, Weng PL, Farida J. Malaysian Journal of Medicine and Health Sciences; 2011;7(1): 27-34.

Supplementary Table 2. Diphtheria tetanus toxoid and pertussis (DTP3) vaccination coverage (%) among 1-year-olds and incidence rate per million population of diphtheria cases in Bangladesh, China, India, Indonesia, Malaysia, Myanmar and the Philippines

Country	Year childhood diphtheria vaccination introduced	Age recommended	DTP3 coverage (%)		Incidence rate per million population in 2010-2019	
			Year 1991	Year 2019	Average annual rate	Highest annual rate
Bangladesh	1985	6, 10, 14 weeks	74	98	0.09	0.22
China	1978	3, 4, 5 months	94	99	0.00	0.00
India	1978	6, 10, 14 weeks	57	91	3.72	7.04
Indonesia	1985	2, 3, 4, 18 months	62	85	2.52	4.80
Malaysia	1964	2, 3, 5, 18 months	92	98	0.36	1.03
Myanmar	1980	2, 4, 6 months	66	90	1.01	2.56
Philippines	1979	6, 10, 14 weeks	90	65	0.59	1.86

Data sources:
World Health Organization. Diphtheria tetanus toxoid and pertussis (DTP3) immunization coverage among 1-year-olds (%) https://www.who.int/data/gho/data/indicators/indicator-details/GHO/diphtheria-tetanus-toxoid-and-pertussis-(dtp3)-immunization-coverage-among-1-year-olds-(-). Accessed 19 September 2020.
World Health Organization. Diphtheria reported cases. Last update: 15-Oct-2020 (data received as of 12-Oct-20). https://apps.who.int/immunization_monitoring/globalsummary/timeseries/tsincidencediphtheria.html. Accessed 9 April 2021.
